# Assembly, Secretory Pathway Trafficking, and Surface Delivery of Kainate Receptors Is Regulated by Neuronal Activity

**DOI:** 10.1016/j.celrep.2017.06.001

**Published:** 2017-06-20

**Authors:** Ashley J. Evans, Sonam Gurung, Kevin A. Wilkinson, David J. Stephens, Jeremy M. Henley

**Affiliations:** 1 School of Biochemistry, Centre for Synaptic Plasticity, Biomedical Sciences Building, University of Bristol, University Walk, Bristol BS8 1TD, UK

**Keywords:** kainate receptor, retention using selective hooks, RUSH, secretory pathway, ER exit sites, Golgi outposts, AMPA receptors, scaling, Q/R editing, ADAR2, PDZ ligand

## Abstract

Ionotropic glutamate receptor (iGluR) trafficking and function underpin excitatory synaptic transmission and plasticity and shape neuronal networks. It is well established that the transcription, translation, and endocytosis/recycling of iGluRs are all regulated by neuronal activity, but much less is known about the activity dependence of iGluR transport through the secretory pathway. Here, we use the kainate receptor subunit GluK2 as a model iGluR cargo to show that the assembly, early secretory pathway trafficking, and surface delivery of iGluRs are all controlled by neuronal activity. We show that the delivery of de novo kainate receptors is differentially regulated by modulation of GluK2 Q/R editing, PKC phosphorylation, and PDZ ligand interactions. These findings reveal that, in addition to short-term regulation of iGluRs by recycling/endocytosis and long-term modulation by altered transcription/translation, the trafficking of iGluRs through the secretory pathway is under tight activity-dependent control to determine the numbers and properties of surface-expressed iGluRs.

## Highlights

• KARs can use a local dendritic secretory network for trafficking to the post-synapse
• Their secretory trafficking is highly activity-dependently regulated
• TTX decreases GluK2 editing, which promotes ER export of new KARs
• KAR activation slows KAR traffic via C-terminal PDZ ligand interactions on GluK2

## Graphical Abstract

**Figure:**
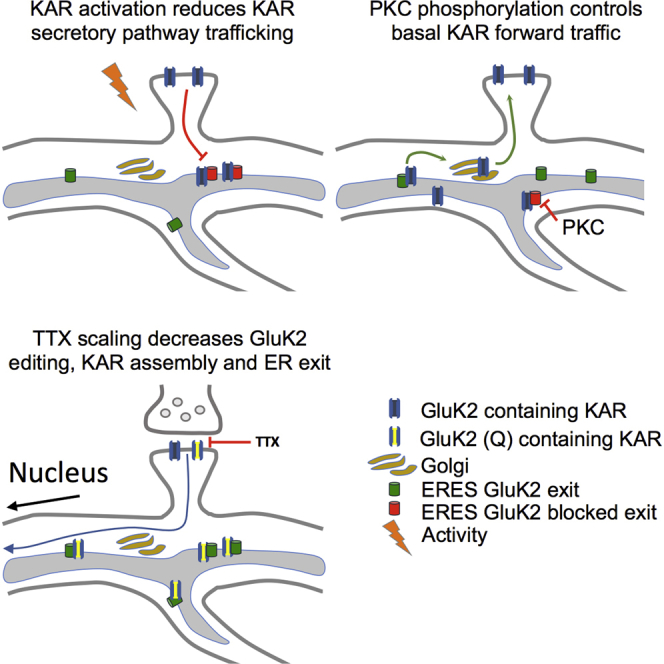

## Teaser

Evans et al. show that secretory pathway trafficking of KARs is highly activity-dependent. This medium-term regulatory mechanism demonstrates how neuronal excitability and network activity are regulated at multiple levels over a range of time courses.

## Introduction

The morphological complexity of neurons presents unique challenges for the timely and appropriate supply of proteins to dynamic and metabolically active synapses. The ionotropic glutamate receptor (iGluR) family comprising N-methyl-D-aspartate (NMDA), α-amino-3-hydroxy-5-methyl-4-isoxazolepropionic acid (AMPA), and kainate receptors (NMDARs, AMPARs, and KARs, respectively) are critical for synaptic transmission and plasticity, and the mechanisms by which iGluRs are delivered to, retained at, and removed from synapses under basal, stimulated, and pathological conditions have been the focus of intense investigation for decades (Huganir and Nicoll, 2013, Granger and Nicoll, 2013, Lerma and Marques, 2013, Henley and Wilkinson, 2016).

The transcription (Liu et al., 2010, Jia et al., 2006, Grooms et al., 2006), RNA editing (Sanjana et al., 2012), translation (Schuman et al., 2006), post-translational modification (Martin et al., 2007, Copits and Swanson, 2013, Lussier et al., 2015, Wilkinson et al., 2012, Chamberlain et al., 2012, Konopacki et al., 2011), and surface endocytosis/recycling (Glebov et al., 2015, Boehm et al., 2006, Palmer et al., 2005, Beattie et al., 2000, Kennedy and Ehlers, 2011) of iGluRs are all activity-dependently regulated. Surprisingly, however, relatively little is known about whether and how the delivery of newly synthesized iGluRs through the secretory pathway is controlled by neuronal activity. Studies using temperature-sensitive vesicular stomatitis virus G transmembrane protein (tsVSV-G) as a cargo marker for the endomembrane system have reported that endoplasmic reticulum (ER) exit sites (ERESs) are both present and utilized in dendrites and that some VSV-G cargo subsequently colocalizes at dendritic Golgi outposts (Torre and Steward, 1996, Presley et al., 1997, Horton and Ehlers, 2003). Using mRNA trafficked from the soma, postsynaptic proteins can be locally translated and post-translationally modified (Cajigas et al., 2012, Holt and Schuman, 2013, Na et al., 2016). Furthermore, transmembrane proteins with an immature glycosylation profile can be surface-expressed, suggesting that not all secretory pathway cargo needs to be processed within the Golgi prior to plasma membrane insertion (Hanus et al., 2016).

Despite its widespread use, the fact that tsVSV-G is an exogenous viral protein and that temperature shifts are required to release it from the ER raise important questions about its fidelity as a reporter for endogenous neuronal proteins. Despite these caveats, neuronal activity can increase VSV-G-containing vesicle delivery through the secretory pathway to the cell surface (Hanus et al., 2014), suggesting, but not directly demonstrating, that the secretory pathway trafficking of endogenous cargos such as iGluRs is likely to be activity-dependently regulated. Furthermore, the secretory pathway trafficking of AMPARs can be regulated by interactions with coat protomer II (COPII) components following activation of metabotropic glutamate receptors (Pick et al., 2017).

To directly monitor iGluR processing and progression through the secretory pathway under basal and stimulated conditions, we adapted the retention using selective hooks (RUSH) system that allows the synchronous release and visualization of the trafficking of cargo through the secretory pathway (Boncompain et al., 2012). We used the KAR subunit GluK2 as a prototypic iGluR cargo. KARs are present at both pre- and postsynaptic membranes, where they perform distinct roles in modulating synaptic transmission, neuronal excitability, and network activity (Contractor et al., 2011, Lerma and Marques, 2013), and they are implicated in processes ranging from neuronal development and differentiation to neurodegeneration and neuronal cell death (Contractor et al., 2011, González-González et al., 2012).

We show that KARs can use a local dendritic secretory pathway. GluK2 editing is activity-dependently controlled, resulting in modulation of KAR assembly (Ball et al., 2010) and subsequent increases in unedited GluK2-containing, calcium-permeable KARs at the cell surface (Egebjerg and Heinemann, 1993). Under basal conditions, the secretory pathway trafficking of GluK2-containing KARs is regulated by protein kinase C (PKC) phosphorylation. In a distinct regulatory process, surface KAR activation slows the progression of newly synthesized KARs through the secretory pathway by modulating interactions at the C-terminal PDZ ligand of GluK2. Together, these data reveal that the delivery of de novo KARs to the cell surface is dynamically regulated in a sophisticated, multilayered manner. These mechanisms provide additional flexibility to neuronal responses to changing cellular environments and network activity.

## Results

### Using RUSH to Assay iGluR Secretory Pathway Trafficking

**Figure 1:**
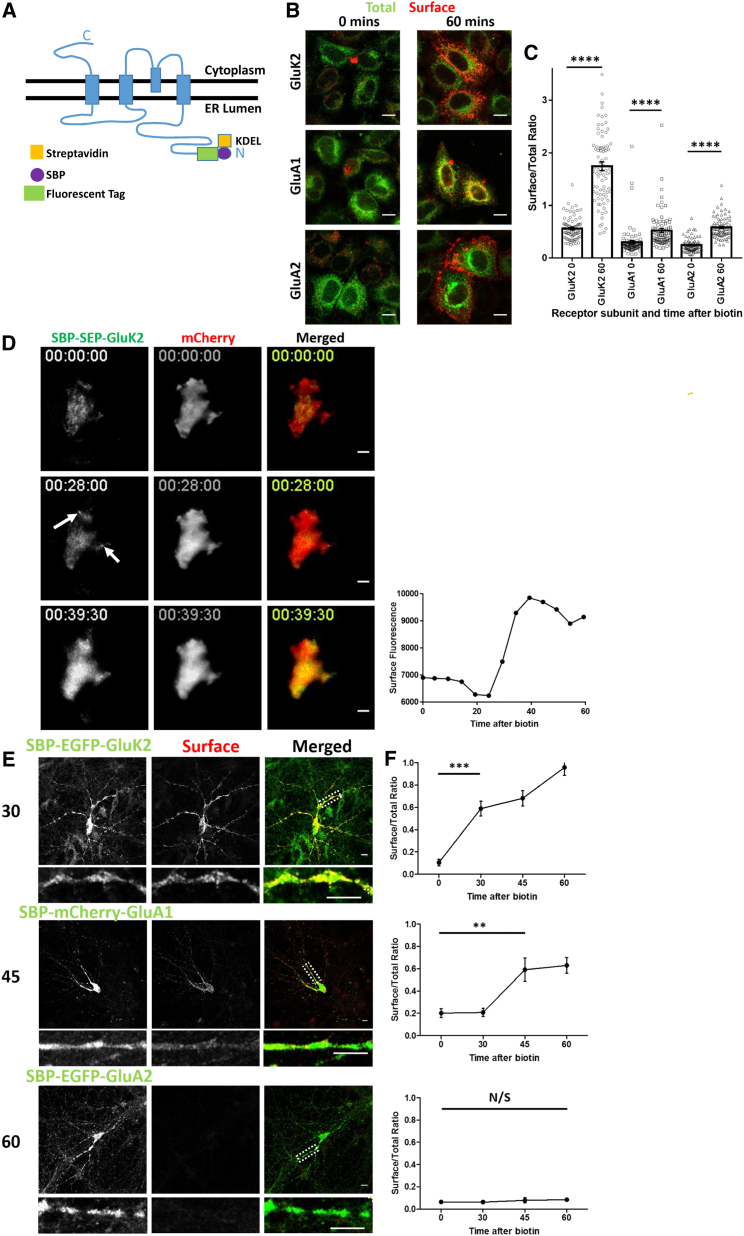
Construction and Validation of RUSH Glutamate Receptor Subunits in HeLa and Primary Hippocampal Neuronal Cells (A) Schematic of a RUSH ionotropic glutamate receptor subunit. SBP, streptavidin-binding peptide. (B) Representative confocal images of the AMPAR subunits SBP-mCherry-GluA1 and SBP-EGFP-GluA2 and the KAR subunit SBP-EGFP-GluK2 in HeLa cells. Receptors are retained in the ER (0 min) and synchronously released by biotin addition, allowing trafficking to the cell surface (60 min after biotin addition). Total, green; surface anti-SBP, red. (C) Quantification of the data represented in (B); three independent experiments, n = 80 cells/condition. ^∗∗∗∗^p < 0.0001, Welch’s t test. (D) Representative still frames of the TIRF microscopy video (Figure S1B; Movie S1), showing the time course of trafficking and analysis of cell surface delivery of SBP-SEP-GluK2 after biotin addition. Arrows indicate sites of exocytosis. Quantification of surface delivery over time is also shown. See also Figure S1B. (E) Representative confocal images of primary hippocampal neurons showing the differential secretory pathway trafficking rates of SBP-EGFP-GluK2, SBP-mCherry-GluA1, and SBP-EGFP-GluA2 containing KARs and AMPARs, respectively. Surface-expressed receptors were visualized using anti-SBP (red) at the indicated times (minutes) after biotin release. White boxes positioned on the merged panels indicate the region of the zoom panel. (F) Quantification of the data shown in (E); three independent experiments, n = 20–24 for each receptor per time point. ^∗∗∗^p < 0.001, ^∗∗^p < 0.01, Welch’s t test. Scale bars, 10 μm.

We utilized the RUSH system by tagging the GluK2 KAR subunit and both the GluA1 and GluA2 AMPAR subunits at the N terminus with a streptavidin-binding peptide (SBP) and a fluorescent tag. When these constructs are coexpressed with a streptavidin-KDEL “hook” that localizes to the lumen of the ER, the SBP-tagged subunits are anchored at the ER membrane (Figure 1A). The retained SBP-tagged receptors can then be synchronously released by biotin addition to monitor their trafficking through the secretory pathway (Figure S1A; Boncompain and Perez, 2012).

We first validated these RUSH constructs in HeLa cells. As expected, the SBP-tagged receptors are efficiently retained in the ER (0 min), and, upon addition of biotin, they are released and move through the secretory pathway, reaching the cell surface after 60 min (Figures 1B and 1C). Interestingly, each of the three different subunits had different kinetics, with much more GluK2 than GluA1 or GluA2 present at the cell surface after 60 min. Because GluK2 traffics most rapidly, we measured the dynamics of surface expression using super-ecliptic pHluorin (SEP)-tagged GluK2 (SBP-SEP-GluK2) (Ashby et al., 2004, Wilkinson et al., 2014) and total internal reflection fluorescence (TIRF) microscopy. GluK2 starts accumulating at the surface ∼30 min after biotin-induced release from the ER (Figure 1D; Figure S1B; Movie S1).

Consistent with the results from HeLa cells, we observed different rates of secretory pathway trafficking for GluK2, GluA1, and GluA2 in hippocampal neurons (Figures 1E and 1F). In agreement with previous reports using endogenous subunits (Greger et al., 2002), SBP-mCherry-GluA1 traffics through the secretory pathway quicker than SBP-EGFP-GluA2. These data show that the RUSH system allows synchronized release of KARs and AMPARs from the ER in both clonal cell lines and neurons and that it provides a powerful tool to investigate the early trafficking steps of iGluRs.

### KARs Use Dendritic ER Exit Sites and Golgi Outposts

**Figure 2:**
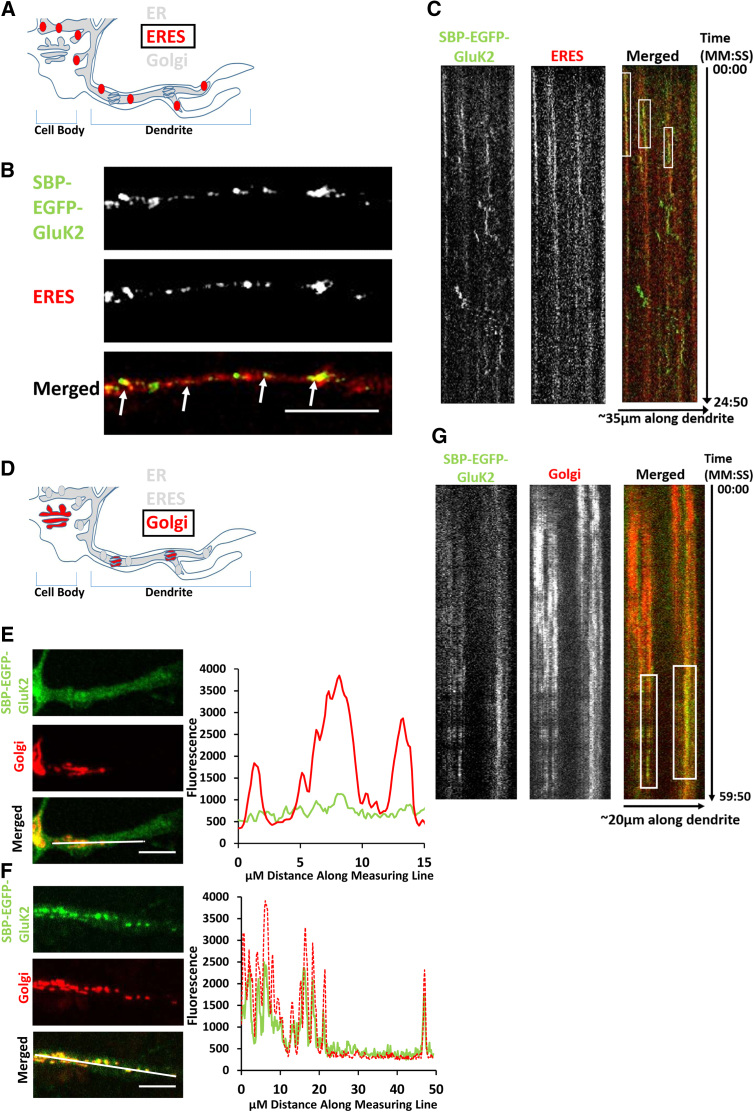
KARs Use Local Secretory Pathway Systems (A) Schematic of dendritic local secretory pathways in neurons, focusing on ER exit sites. (B) Representative fixed confocal images of dendritic ERESs (using the marker mRuby-Sec23a) and SBP-EGFP-GluK2 10 min after biotin addition. White arrows in the merged panel indicate colocalization. (C) Kymograph (Movie S2) of SBP-EGFP-GluK2 and mRuby-Sec23a up to 24 min 50 s after biotin addition, with a frame being taken every 10 s. White boxes on the merged panel show the duration of colocalization. (D) Schematic of dendritic local secretory pathways in neurons, focusing on the Golgi. (E) Representative fixed confocal images of SBP-EGFP-GluK2 colocalization with the Golgi marker Sialyltransferase-mCherry (Golgi) before biotin release and a line trace illustrating lack of colocalization along the line indicated in white in the merged image. (F) Representative fixed confocal images of SBP-EGFP-GluK2 30 min after biotin-induced release with Sialyltransferase-mCherry (Golgi) and line trace quantification. (G) Kymograph (Movie S3) of SBP-EGFP-GluK2 and GalT-mCherry (Golgi) after biotin addition up to 59 min 50 s, with a frame being taken every 10 s. White boxes on the merged panel show colocalization duration. Scale bars, 10 μm.

VSV-G and NMDARs have been reported to use dendritic ERESs and Golgi outposts (Figures 2A and 2D) for post-translational modification, which is facilitated by the interacting proteins CASK and SAP97 (Horton and Ehlers, 2003, Jeyifous et al., 2009). Although KARs bind to both CASK and SAP97 through a PDZ ligand/domain interaction (Coussen et al., 2002, Hirbec et al., 2003), it is unknown whether KARs use local secretory pathways. We therefore investigated this using SBP-EGFP-GluK2 in neurons. SBP-EGFP-GluK2 colocalizes with mRuby-Sec23A-labeled ERESs (Budnik and Stephens, 2009, Hughes and Stephens, 2010) in dendrites after biotin-induced release (Figures 2B and 2C; Figure S2A; Movie S2).

VSV-G colocalizes with local Golgi outposts after release from dendritic ERESs (Horton and Ehlers, 2003; Figure 2D). Before release, SBP-EGFP-GluK2 is retained in the ER and does not colocalize with dendritic Golgi outposts (Figure 2E). However, 30 min after ER release by biotin, SBP-EGFP-GluK2 strongly colocalized at Golgi outposts (Figures 2F and 2G; Figure S2B; Movie S3), demonstrating that KARs utilize local secretory pathway systems.

### Assembly and Surface Delivery of Newly Synthesized KARs Is Controlled by Chronic Changes in Synaptic Activity and Mediated by Changes in the RNA Editing of GluK2

**Figure 3:**
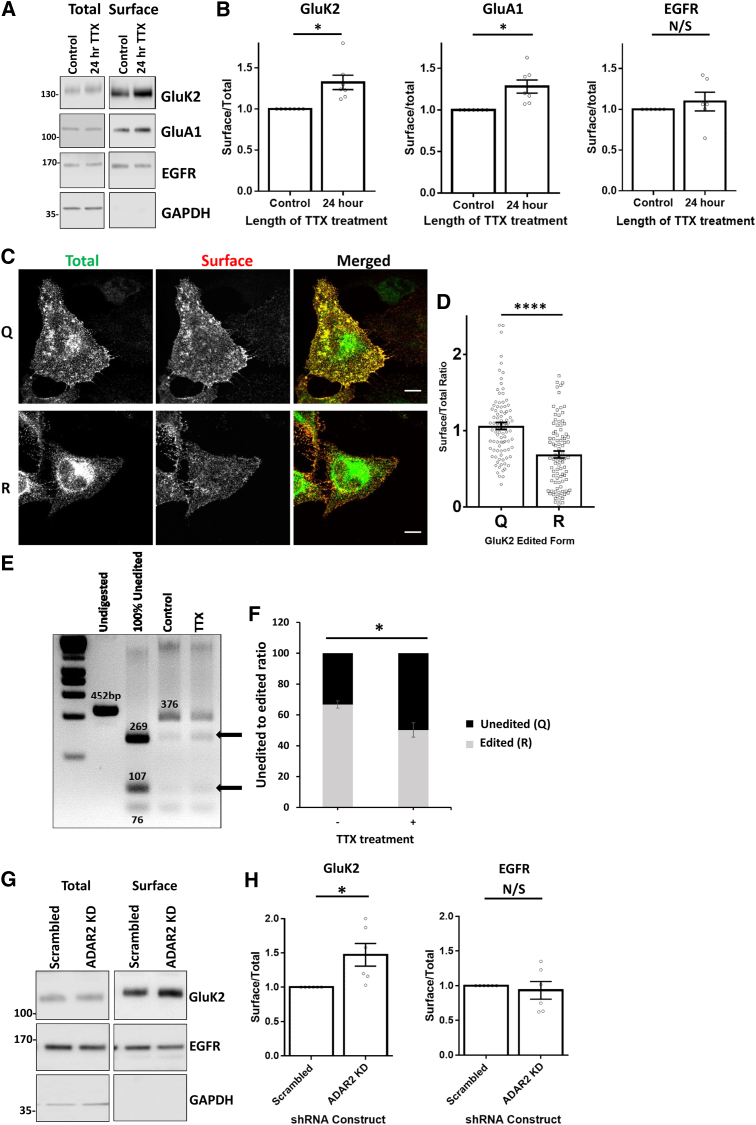
KAR ER Exit Is Regulated by Activity-Dependent Changes in RNA Editing of GluK2 (A) Representative western blots of surface-biotinylated KAR and AMPAR subunits and EGFR in hippocampal neurons. The blots show surface and total levels of subunits with or without 24-hr treatment with 1 μM TTX to suppress synaptic activity. (B) Quantification of immunoblots and comparison of surface-to-total ratios from six (GluA1 and EGFR) and seven (GluK2) independent experiments. ^∗^p < 0.05, Wilcoxon matched pairs signed-rank test. (C) SBP-EGFP-GluK2 unedited (Q) or edited (R) RUSH constructs transfected into HeLa cells with addition of biotin at the time of transfection to allow basal expression. Surface RUSH KARs were labeled with anti-SBP for a duration of 5 min. See also Figure S3A. (D) Quantification from (C), representative of three independent experiments (n = 90). ^∗∗∗∗^p < 0.0001, Welch’s t test. (E) RT-PCR and digestion analysis of levels of unedited and edited GluK2 with or without TTX treatment. Black arrows indicate unedited forms of GluK2. (F) Quantification of unedited and edited GluK2 with or without TTX treatment (n = 5). ^∗^p < 0.05, Welch’s t test. (G) Representative western blots of surface-biotinylated GluK2 and EGFR after lentiviral infection of primary hippocampal neurons with either scrambled or ADAR2-targeting shRNA. The blots show both total and surface levels of GluK2 and EGFR after 5 days of knockdown. See also Figure S3B. (H) Quantification of immunoblots and comparison of surface-to-total ratios from six independent experiments. ^∗^p < 0.05, Wilcoxon matched pairs signed-rank test.

NMDAR and AMPAR surface expression scales in response to chronic down- or upregulation of synaptic activity (Rao and Craig, 1997, Shepherd et al., 2006, Turrigiano, 2012). To address whether KARs also scale, we chronically suppressed synaptic activity in hippocampal neurons with tetrodotoxin (TTX) for 24 hr. As expected, TTX significantly increased GluA1-containing AMPAR surface expression and also increased GluK2-containing KARs at the cell surface with no change in surface epidermal growth factor receptors (EGFRs) (Figures 3A and 3B), indicating that chronic blockade of activity upscales GluK2-containing KARs.

The pre-mRNAs encoding GluA2 and GluK2 can undergo editing at a site within the channel pore that changes a glutamine (Q) residue to an arginine (R) in the translated subunit (Sommer et al., 1991). This Q/R editing alters the calcium permeability of surface-expressed AMPARs (Burnashev et al., 1992) and KARs (Köhler et al., 1993). Q/R editing also regulates AMPAR and KAR subunit assembly and ER exit (Greger et al., 2002, Ball et al., 2010). We generated RUSH variants of edited and unedited GluK2, and, consistent with previous observations (Ball et al., 2010), the edited (R) form of SBP-EGFP-GluK2 exhibited lower levels of surface expression compared with the unedited Q form in HeLa cells after 24 hr of biotin-induced release (Figures 3C and 3D; Figure S3A). Furthermore, TTX decreases Q/R editing of GluK2 (Figures 3E and 3F). We hypothesized that this change in GluK2 editing will promote KAR assembly and ER exit, resulting in increased surface expression. To test this, we knocked down ADAR2, the enzyme responsible for GluK2 editing (Nishikura, 2016; Figure S3B). ADAR2 knockdown reduced GluK2 editing similar to TTX treatment (Figure S3C) and upscaled surface GluK2 in the absence of TTX (Figures 3G and 3H), indicating that KAR scaling is mediated, at least in part, by activity-dependent regulation of GluK2 Q/R editing.

### PKC Phosphorylation Regulates Basal KAR Trafficking through the Secretory Pathway

**Figure 4:**
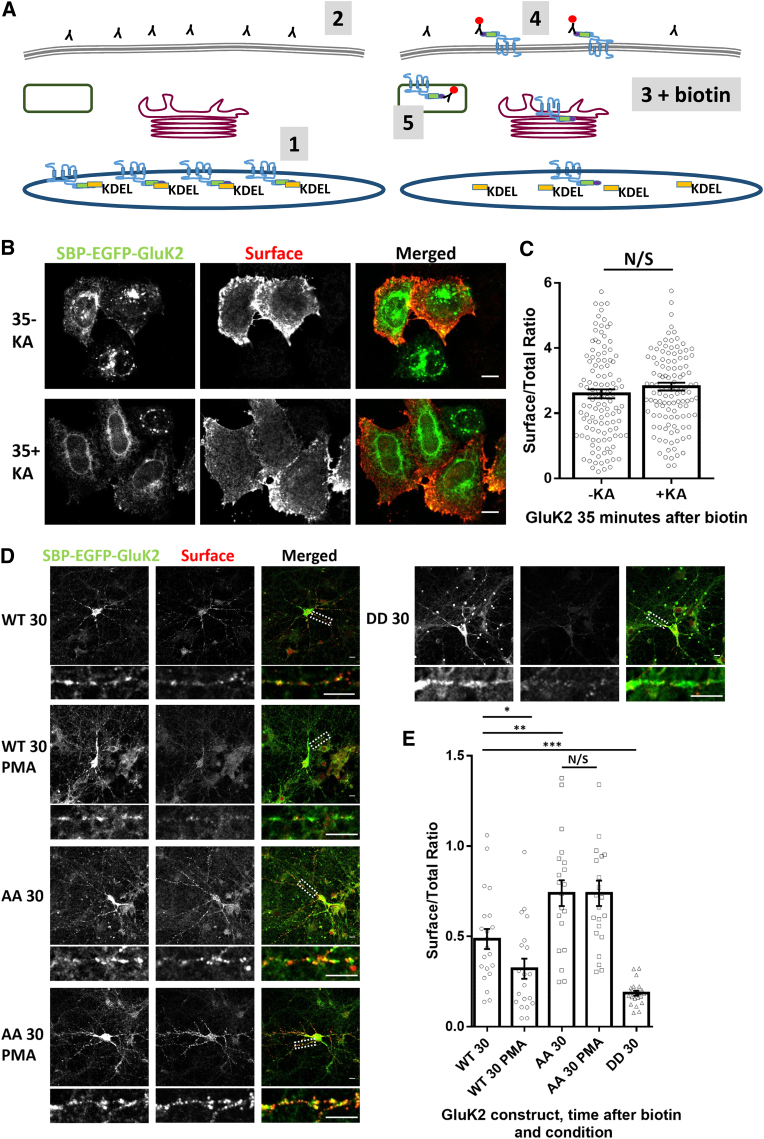
Basal PKC Phosphorylation Suppresses Surface Delivery of KARs (A) Schematic illustrating the live labeling protocol used to exclude any contribution of changes in endocytosis. 1: hooked RUSH receptor before the addition of biotin. 2: live label with anti-SBP antibody. 3: addition of biotin allows release of receptors and accumulation at the cell surface. 4: anti-SBP antibodies bind to newly exposed SBP-tagged receptors. 5: a proportion of receptors internalize, but cells are permeabilized and labeled with a secondary antibody labeling all receptors that have been surface-exposed. (B) Representative images of the live labeling protocol showing that 100 μM KA does not change the secretory pathway delivery and extent of surface expression of SBP-EGFP-GluK2 in HeLa cells 35 min after biotin addition. See also Figure S4A. (C) Quantification of the data shown in (B); two independent experiments, n = 80. p > 0.05, Welch’s t test. (D) Representative images of hippocampal neurons expressing SBP-EGFP-GluK2 WT, SBP-EGFP-GluK2 S846A/S868A, or SBP-EGFP-GluK2 S846D/S868D 30 min after biotin in the presence or absence of PMA. Total receptor distribution was measured using the EGFP tag, and surface-expressed receptors were determined using live labeling with anti-SBP. See also Figure S4B. (E) Quantification of three independent experiments (n = 17–22). ^∗∗∗^p < 0.0005, ^∗∗^p < 0.01, ^∗^p < 0.05; Welch’s t test. Scale bars, 10 μm.

To measure the secretory pathway trafficking and surface expression of de novo KARs without confounding issues from endocytosis and recycling of KARs, we modified the RUSH protocol to measure all subunits that reach the cell surface by live labeling (Figure 4A). To ensure that this live labeling protocol faithfully reports only secretory pathway trafficking to the cell surface and is not affected by rates of endocytosis, we exposed SBP-EGFP-GluK2-expressing HeLa cells to kainate to promote KAR endocytosis (Figure S4A). Comparable surface delivery levels of SBP-EGFP-GluK2 were observed with and without kainate, confirming that this procedure only reports de novo KARs delivered by the secretory pathway (Figures 4B and 4C).

Serines 846 and 868 in the C terminus of GluK2 are phosphorylated by PKC, and phosphomimetic mutations of these residues cause ER retention (Konopacki et al., 2011). We therefore used the RUSH assay to assess the role of GluK2 phosphorylation in KAR trafficking through the secretory pathway. We mutated both S846 and S868 to non-phosphorylatable alanines (SBP-EGFP-GluK2-AA) or to phosphomimetic aspartic acid residues (SBP-EGFP-GluK2-DD). As predicted, the AA phospho-null mutant traffics more and the DD phosphomimetic mutant traffics less efficiently to the cell surface than the wild-type SBP-EGFP-GluK2 under non-stimulated conditions. In parallel, we tested the effect of the PKC activator phorbol 12-myristate 13-acetate (PMA) on surface accumulation. Consistent with the mutant data, PMA decreased the surface expression of SBP-EGFP-GluK2, whereas SBP-EGFP-GluK2-AA was unaffected (Figures 4D and 4E; Figure S4B). Our interpretation of these results is that a proportion of GluK2 is basally phosphorylated by PKC in the ER and that this provides a mechanism to regulate the ER exit and supply of de novo KARs for delivery to the cell surface.

### Activation of Surface-Expressed KARs Regulates De Novo KAR Delivery to the Cell Surface

**Figure 5:**
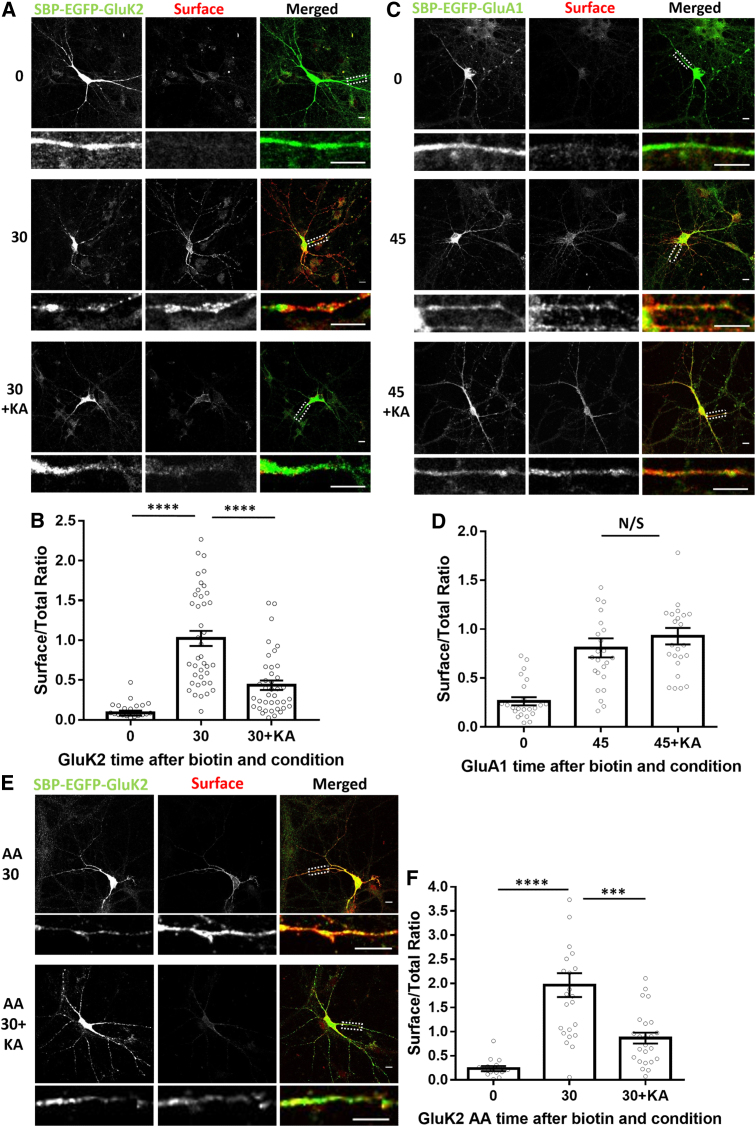
KAR Progress through the Secretory Pathway Is Regulated by Transient KAR Stimulation (A) Representative images of SBP-EGFP-GluK2 without biotin (0), with biotin for 30 min (30), or with a transient 5-min pre-treatment with 10 μM kainate before biotin addition (30+KA). Total SBP-EGFP-GluK2 was visualized with EGFP, and the surface-expressed SBP-EGFP-GluK2 was live-labeled with anti-SBP. (B) Quantification of five independent experiments (n = 24–40). ^∗∗∗∗^p < 0.0001, Welch’s t test. (C) Representative images of SBP-EGFP-GluA1 without biotin (0), with biotin for 45 min (45), or with a transient 5-min pre-treatment with 10 μM kainate before biotin addition (45+KA). Total SBP-EGFP-GluA1 was visualized with EGFP, and the surface-expressed SBP-EGFP-GluA1 was live-labeled with anti-SBP antibody. (D) Quantification of three independent experiments; n = 24 for all conditions. p > 0.05, Welch’s t test. (E) Representative images of SBP-EGFP-GluK2 S846A/S868A, 30 min after biotin (AA 30), or with a transient 5-min pre-treatment with kainate before biotin addition (AA 30+KA). Total SBP-EGFP-GluK2 S846A/S868A was visualized with EGFP, and surface-expressed SBP-EGFP-GluK2 S846A/S868A was live-labeled with anti-SBP antibody. (F) Quantification of three independent experiments; n = 15–30. ^∗∗∗∗^p < 0.0001, ^∗∗∗^p < 0.001, Welch’s t test. Scale bars, 10 μm.

Our data demonstrate that, rather than being a constitutive process, the secretory pathway trafficking of KARs is subject to strict regulation. Under basal conditions, PKC phosphorylation limits the supply of GluK2-containing KARs, and chronic suppression of synaptic activity reduces Q/R editing, which promotes KAR assembly and ER exit. Therefore, we next tested the effects of direct activation of surface-expressed KARs on SBP-EGFP-GluK2 trafficking. We used our previously described transient kainate application protocol (5 min, 10 μM kainate + TTX followed by washout), which increases KAR surface expression (Martin et al., 2008, González-González and Henley, 2013). This transient kainate application prior to biotin-induced SBP-EGFP-GluK2 release from the ER caused a significant reduction in trafficking through the secretory pathway to the cell surface (Figures 5A and 5B). In contrast, the secretory pathway trafficking of SBP-EGFP-GluA1 was unaffected (Figures 5C and 5D). These results indicate that transient activation of surface KARs can selectively reduce the trafficking of de novo KARs through the secretory pathway to control the supply of receptors available for insertion at the cell surface (Figure S5). We initially hypothesized that phosphorylation of S846 and S868 may mediate the kainate-evoked reduction in the surface delivery of de novo KARs (Nasu-Nishimura et al., 2010, Konopacki et al., 2011). Contrary to our expectations, however, secretory pathway trafficking of the non-phosphorylatable SBP-EGFP-GluK2-AA was also significantly reduced by transient kainate stimulation (Figures 5E and 5F). Thus, kainate regulation of KAR secretory pathway trafficking appears to be mediated via a mechanism other than PKC phosphorylation.

### The GluK2 PDZ Ligand Is Involved in Basal and Activity-Dependent Delivery of De Novo Receptors to the Cell Surface

The GluK2 PDZ ligand (^905^ETMA^908^) interacts with multiple PDZ domain-containing proteins, including SAP97, PICK1, PSD95, GRIP, syntenin, and CASK (Coussen et al., 2002, Hirbec et al., 2002), and inhibition of PDZ interactions using a competing peptide leads to a rundown in KAR excitatory postsynaptic currents (EPSCs) (Hirbec et al., 2003). We therefore mutated the extreme C-terminal PDZ ligand of SBP-EGFP-GluK2 from the wild-type sequence ETMA to EPAS, which cannot interact with PDZ domain-containing proteins (Hirbec et al., 2003).

**Figure 6:**
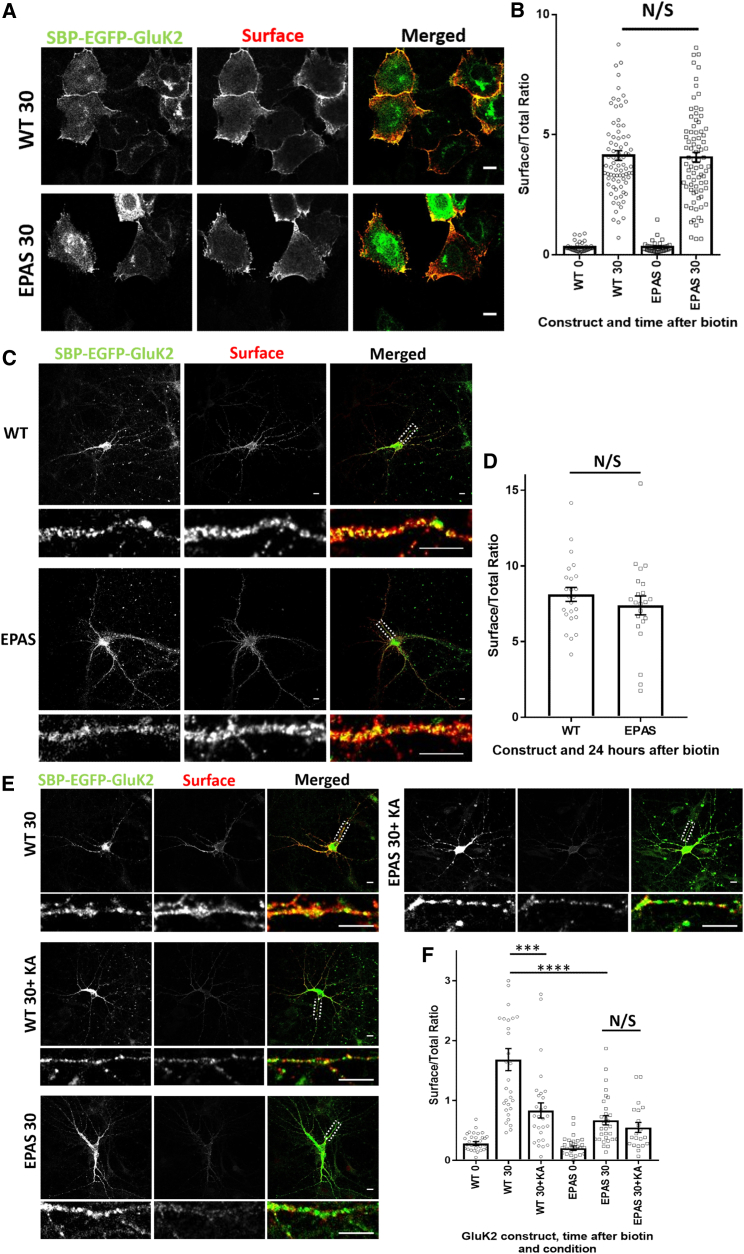
The PDZ Ligand of GluK2 Is Involved in Both Basal and Activity-Dependent Progression of KARs through the Secretory Pathway (A) Representative images of HeLa cells showing the distributions of exogenously expressed SBP-EGFP-GluK2-ETMA or SBP-EGFP-GluK2-EPAS before and 30 min after addition of biotin. In all cases, EGFP was used to visualize total receptors, and surface-expressed KARs were visualized by live labeling with anti-SBP antibody. (B) Quantification of two independent experiments; n = 40 for 0 and n = 80 for 30. p > 0.05, Welch’s t test. See also Figure S6A. (C) Representative images of hippocampal neurons expressing SBP-EGFP-GluK2-ETMA or SBP-EGFP-GluK2-EPAS 24 hr after addition of biotin. EGFP was used to visualize total receptors, and surface-expressed KARs were visualized by live labeling with anti-SBP antibody. (D) Quantification of three independent experiments; n = 22–24. p > 0.05, Welch’s t test. (E) Representative images of hippocampal neurons expressing SBP-EGFP-GluK2-ETMA or SBP-EGFP-GluK2-EPAS before or 30 min after addition of biotin, with or without a transient 5-min pre-treatment of 10 μM kainate. EGFP was used to visualize total receptors, and surface-expressed KARs were visualized by live labeling with anti-SBP antibody. See also Figure S6B. (F) Quantification of four independent experiments; n = 21–32. ^∗∗∗∗^p < 0.0001, ^∗∗∗^p < 0.001, Welch’s t test. Scale bars, 10 μm.

In HeLa cells, secretory pathway trafficking for both wild-type SBP-EGFP-GluK2-ETMA and the PDZ ligand mutant SBP-EGFP-GluK2-EPAS was comparable (Figures 6A and 6B; Figure S6A). Similarly, there was no difference between the wild-type and EPAS mutant when expressed in neurons with addition of biotin at the same time as transfection to allow continuous release of the receptor from the ER to determine their steady-state localization (Figures 6C and 6D; Figure S6B).

We next performed experiments corresponding to those shown in Figure 5, where biotin was added to elicit synchronized release of SBP-EGFP-GluK2 or SBP-EGFP-GluK2-EPAS with or without a transient kainate stimulation prior to biotin application. This transient kainate stimulation significantly decreased the secretory pathway trafficking of wild-type SBP-EGFP-GluK2-ETMA (Figures 6E and 6F). Interestingly, the secretory pathway trafficking of SBP-EGFP-GluK2-EPAS was significantly reduced compared with SBP-EGFP-GluK2-ETMA under basal conditions. Furthermore, the secretory pathway trafficking of SBP-EGFP-GluK2-EPAS was not further decreased by kainate application, indicating that preventing PDZ interactions occludes the kainate-induced reduction in secretory pathway trafficking (Figures 6E and 6F; Figure S6B). Together, these results demonstrate that, although the GluK2 PDZ interactions do not affect the steady-state localization of GluK2-containing KARs, they regulate their activity-dependent secretory pathway trafficking.

## Discussion

Here we show that GluK2-containing KARs use a local secretory pathway system close to their sites of membrane delivery. Rather than being a constitutive process, KAR traffic through the secretory pathway is tightly and differentially regulated under chronically suppressed, basal, and transiently stimulated conditions.

### Validation of the RUSH System in Neurons

RUSH provides a powerful system for investigating the dynamics of AMPAR and KAR trafficking to the cell surface. We show that the RUSH GluA1 and GluA2 AMPAR subunits and GluK2 KAR subunit are effectively retained at the ER membrane and can be synchronously released on demand by addition of biotin in both clonal cells and primary neurons. Importantly, the rates of traffic through the secretory pathway we measured for GluA1 and GluA2 agree well with rates reported for endogenous AMPAR subunits monitored by pulse-chase radiolabeling, with GluA1 trafficking more rapidly than GluA2 (Greger et al., 2002, Greger and Esteban, 2007).

### KAR Scaling Is Mediated by GluK2 Editing

**Figure 7:**
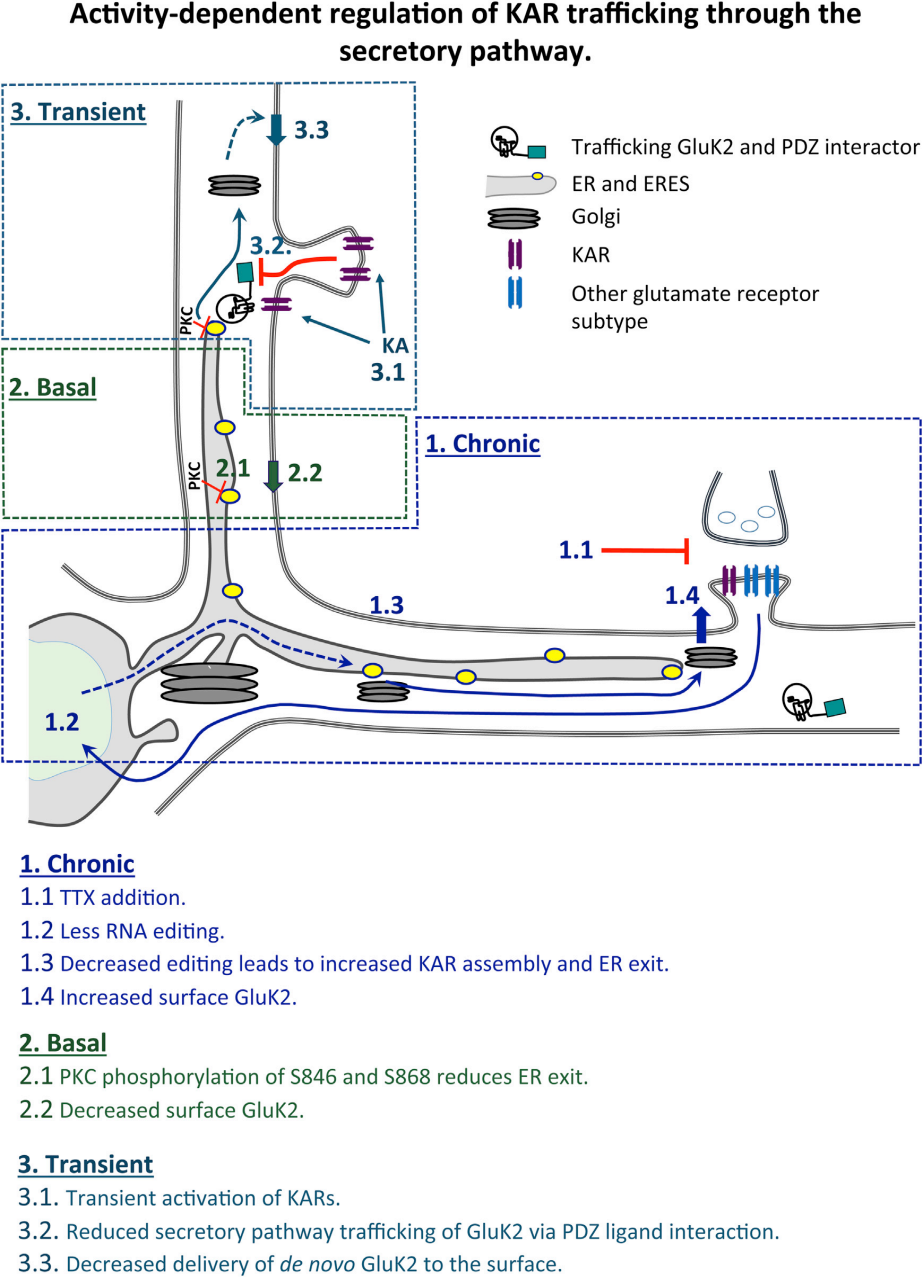
Model Shown is a schematic summarizing our results.

AMPARs and NMDARs scale in response to a prolonged decrease or increase in synaptic activity (Turrigiano, 2012). Given their importance in neuronal circuit development and both pre- and postsynaptic function (Contractor et al., 2011, Lerma and Marques, 2013) we reasoned that it is likely that KARs also need to be tuned in response to overall activity changes. Consistent with this, KARs are scaled by chronic suppression of synaptic activity (Yan et al., 2013). We propose a mechanism analogous to NMDAR scaling whereby changes in RNA editing regulate the ER exit and, consequently, the availability of new NMDARs for delivery to the surface (Mu et al., 2003). We show that chronic suppression of synaptic activity decreases Q/R editing of GluK2, which promotes KAR assembly, ER exit, and delivery to the cell surface (Figure 7). This mechanism to restrict the amount of KARs reaching the cell surface likely plays a key role in controlling neuronal excitability, and it is notable that transgenic mice deficient in Q/R editing display increased seizure vulnerability (Vissel et al., 2001).

### PKC Phosphorylation of GluK2 Controls Basal Trafficking through the Secretory Pathway

Agonist activation of surface-expressed KARs causes PKC phosphorylation of GluK2 at S846 and S868, which promotes SUMOylation and KAR endocytosis (Martin et al., 2007, Konopacki et al., 2011, Chamberlain et al., 2012). Furthermore, phosphomimetic serine-to-aspartate mutations of residues 846 and 868 in GluK2 impede KAR traffic to the cell surface (Nasu-Nishimura et al., 2010, Konopacki et al., 2011). Here we show that these PKC phosphorylation sites are involved in ER exit of KARs and that preventing PKC phosphorylation of GluK2 by mutating S846 and S868 to alanine increases basal rates of secretory pathway trafficking. Thus, PKC phosphorylation of S846 and S868 in GluK2 exerts multiple levels of control over KAR trafficking, including regulating the number of GluK2-containing KARs that can exit the ER and enter the secretory pathway (Figure 7).

### Transient Kainate Receptor Activation Downregulates the Delivery of Newly Synthesized KARs

Transient KAR activation can elicit a lasting upregulation of KARs at the cell surface because of increased recycling back to the surface (Martin et al., 2008, González-González and Henley, 2013), and this form of KAR activation can also induce long-term potentiation (LTP) of AMPARs (Petrovic et al., 2017, Sanjana et al., 2012). Here we show that transient kainate stimulation decreases the supply of de novo KARs through the secretory pathway. We interpret these results to indicate a negative feedback mechanism that can limit the extent of the increase in KAR surface expression. Thus, following a kainate-induced increase in surface expression of locally available KARs, the supply of new receptors is restricted to prevent positive feedback, leading to further increases in KAR surface expression and uncontrolled neuronal excitability and excitotoxicity. This agonist-mediated regulation of KAR secretory pathway trafficking is not due to changes in the phosphorylation status of S846 and S868 because secretory pathway traffic of the PKC non-phosphorylatable GluK2 mutant was also reduced by transient kainate application. Furthermore, this regulatory system is KAR-specific because kainate stimulation does not regulate the secretory pathway traffic of AMPARs.

### GluK2 PDZ Interactions and the Activity-Controlled Secretory Pathway

The PDZ ligand of GluK2 binds to an array of interacting proteins, including PSD95, SAP97, PICK1, GRIP, CASK, and syntenin (Coussen et al., 2002, Hirbec et al., 2003) that control many aspects of KAR localization and function. C-terminal truncations of GluK2 that removed the PDZ ligand did not result in major defects in trafficking in heterologous cells, indicating that the PDZ ligand is not required for folding or ER exit (Yan et al., 2004). Consistent with this, secretory pathway trafficking is similar for GluK2 containing either the wild-type (ETMA) or mutated non-binding (EPAS) PDZ ligand. Furthermore, the steady-state localization of PDZ ligand mutants was also unchanged, suggesting that, although PDZ interactions are important for the dynamics of secretory pathway trafficking, they are not required for correct localization of GluK2.

Disruption of the GluK2 PDZ ligand did, however, significantly decrease basal secretory pathway trafficking in neurons, which occluded the kainate-induced reduction of secretory pathway trafficking. This is consistent with our previous observation that a peptide corresponding to the PDZ ligand of GluK2 can out compete endogenous interactions and, consequently, causes rundown of KAR-mediated EPSCs (Hirbec et al., 2003) and long-term depression of kainate receptor-mediated synaptic transmission (Park et al., 2006). Both of these reductions in KAR-mediated transmission are sustained over long periods, and we propose that they are attributable, at least in part, to the activity-dependent reductions in KAR secretory pathway trafficking we describe here.

In summary, the secretory pathway trafficking of KARs occurs through local secretory pathways using ERES in distal dendrites and Golgi outposts. We note, however, that it has recently been reported that KARs with an immature glycosylation state accumulate at the cell surface, suggesting that not all KARs are processed within the Golgi (Hanus et al., 2016). Like long-term regulation of iGluR synthesis by transcription and translation and short-term regulation by endocytosis and recycling of surface-expressed iGluRs, the intermediate-term processes of trafficking through the secretory pathway are also under tight activity-dependent control. These additional medium-term regulatory mechanisms add further flexibility and subtlety to neuronal excitability and network activity. Consistent with this general idea, the secretory pathway trafficking of the GluA2 AMPAR subunit can be regulated by an activity-dependent interaction with COPII vesicle proteins during mGluR-mediated, long-term depression (Pick et al., 2017). These findings open exciting avenues of research into how defects in this local secretory trafficking of KARs contribute to diseases such as epilepsy and autism, in which misregulation of KARs have been strongly implicated.

## Experimental Procedures

### Primary Neuronal Culture

Embryonic hippocampal neurons were isolated from E18 Wistar rats as described previously (Martin and Henley, 2004). The cells were then plated out at various densities and cultured for up to 2 weeks. Plating medium was left on the cells for the first 24 hr: Neurobasal (Gibco) medium supplemented with horse serum (10%), GS21 (GlobalStem), and 2 mM Glutamax. Then this was changed to feeding medium (lacking horse serum) for the duration of the culture. Animal care and all experimental procedures were conducted in accordance with UK Home Office and University of Bristol guidelines.

### DNA Construct Generation and Transfection

All RUSH iGluR constructs were assembled in the RUSH vector system as described previously (Boncompain and Perez, 2013). Briefly, glutamate receptors were cloned so that the fluorescent protein (FP) and the SBP were positioned immediately after an N-terminal signal peptide (in all cases, the interleukin-2 signal peptide was used). The structure is therefore SP-SBP-FP-glutamate receptor. All GluK2 constructs had an additional myc tag on the N terminus, and all SBP-EGFP-GluK2 constructs used, unless specified otherwise, were Q-edited versions (Martin et al., 2007). The R-edited version of SBP-EGFP-GluA2 was used throughout. For each iGluR construct, a forward primer was designed with an FseI restriction site and the reverse primer with a PacI site, followed by cloning of the PCR product using standard molecular biology techniques. QuikChange mutagenesis was used to introduce point mutations. Fluorescent tags were switched using forward and reverse primers with SbfI and FseI sites, respectively. DH5α was used to clone and amplify DNA. A Lipofectamine 2000 method from Invitrogen was used to introduce DNA into hippocampal neurons (days in vitro [DIV] 13–14) and HeLa cells. Cells were incubated at 37°C and 5% CO_2_ for 18–24 hr before fixation or live imaging (Boncompain et al., 2012).

### Virus Generation and shRNA

For ADAR2 knockdown experiments, a short hairpin RNA (shRNA)-targeting ADAR2 (target sequence AACAAGAAGCTTGCCAAGGCC) under the control of an H1 promoter was cloned into a modified form of the lentiviral vector pXLG3. Lentiviruses were then produced using HEK293T cells, harvested, and added to DIV 9/10 hippocampal cells for 5 days, followed by surface biotinylation (Rocca et al., 2017).

### Live Cell Surface Labeling and Fixation

All experiments with surface staining were performed using a live imaging protocol. RUSH-transfected hippocampal neurons or HeLa cells were live-labeled using an anti-SBP (Millipore, monoclonal, clone 20, MAB10764) primary antibody.

For activity experiments, cells were incubated for 5 min in 1 μM TTX with or without 10 μM kainate (Tocris Bioscience) and then washed with PBS. GYKI53655 (40 μM, Abcam) was included to block AMPARs. The PKC activator PMA (12-O-tetradecanoylphorbol-13-acetate [TPA], Cell Signaling Technology) was used at 1 μM, and DMSO was used as a vehicle. HEPES-buffered saline (HBS) (NaCl, 140 mM; KCl, 5mM; glucose, 15 mM; HEPES, 25 mM; CaCl_2_, 1.5 mM; and MgCl_2_, 1.5 mM) containing D-biotin (40 μM, Sigma) was added to the cells in the presence of the anti-SBP antibody (1/500 dilution) for different times. The 0 time point was incubated with just anti-SBP and no biotin but always for the longest time being tested. After completion, cells were washed with PBS multiple times before fixing in 4% paraformaldehyde (PFA) for 8–10 min and quenched in 100 mM glycine (Severn Biotech). Cells were then incubated with PBS + 3% BSA (Sigma) with 0.1% Triton X-100 (Fisher Scientific) for 10 min and then with PBS + 3% BSA for a further 10 min.

For all non-live labeling experiments, the medium was removed from cells on the day of fixation. The cells were then washed with PBS, and methanol (−20°C) was added to the cells and incubated at −20°C for 4 min. The cells were then washed in PBS.

### Fixed Immunostaining and Secondary Antibody Labeling

After fixation, the cells were washed in PBS before addition of primary antibodies diluted in PBS + 3% BSA for an incubation time of 60 min. Cells were washed in PBS before adding secondary antibodies (Jackson ImmunoResearch Laboratories), which were used at 1:400 dilution in PBS + 3% BSA. The cells were then washed again in PBS and mounted onto glass slides using Fluoromount-G with DAPI (eBioscience).

### Imaging and Analysis

A Leica SP5 confocal microscope was used to image both the total (EGFP/mCherry) and surface (anti-SBP) fluorescence, and a surface-to-total ratio was calculated after analysis. ImageJ was used to analyze surface-to-total ratios. Multiple boxes were drawn onto proximal and secondary dendrites that had an EGFP/mCherry signal present. The total fluorescence was measured, and then the channel was switched to surface fluorescence to measure the surface. A surface-to-total ratio was measured, and then an average of the multiple box measurements gave a cell surface-to-total ratio. At least eight cells were analyzed per experiment, and experiments were repeated at least three times using cells from independent dissections. TIRF analysis was done using the mCherry signal to mark the cell surface. ImageJ was used to then measure the accumulation of SEP fluorescence. Prism and either Welch’s t tests (direct comparison of two time points/conditions/receptors) or Wilcoxon matched-pairs signed-rank (normalized control sample) tests were used to determine statistical significance. Data are represented as mean, and SEM values are used for error bars. Kymograph live imaging representations and colocalization line traces were made using ImageJ. The scale bars used throughout all figures represent 10 μm.

### RUSH Wide-Field Imaging

A Nikon Ti microscope with a Plan Apo VC 60× oil differential interference contrast (DIC) lens and an Andor DU-885 camera were used to acquire live wide-field RUSH images. The heated stage was pre-heated to 37°C. The cell medium was replaced with 1 mL pre-warmed HBS. When RUSH-transfected cells were found, biotin was added to the cells by diluting biotin to a 2× working solution in HBS. 1 mL of this 2× biotin working solution was added to the original 1 mL of imaging medium already on the cells. The cells were imaged over time periods of up to 60 min, with a frame being taken every 5 or 10 s.

### RUSH TIRF Imaging

A Leica AM TIRF MC system attached to a Leica DMI 6000 inverted epifluorescence microscope was used to image the surface of cells. A 63× oil lens was used. HBS was added to the cells, and the cells were found and focused, and the cell surface plane (mCherry) was found using automated TIRF angles. Frames were taken every 30 s.

### Scaling, Surface Biotinylation, and Western Blot

Hippocampal neurons (DIV 14–15) plated at a density of 500,000 per well of a 6-well dish were treated with 1 μM TTX for 24 hr. All steps were performed on ice with ice-cold buffers unless stated otherwise. After the stated treatments, hippocampal neurons were washed twice in PBS. Surface proteins were labeled with membrane-impermeable Sulfo-NHS-SS biotin (0.3 mg/mL, Thermo Scientific) for 10 min on ice and washed three times with PBS. 50 mM NH_4_Cl was added to quench free biotin-reactive groups, and cells were extracted with lysis buffer (50 mM Tris [pH 7.4], 150 mM NaCl, 1% Triton, 0.1% SDS, and protease inhibitor [Complete, Roche]), incubated on ice for 30 min, and centrifuged (15,000 × *g*, 4°C, 20 min) to remove insoluble cell debris. For isolation of surface proteins, samples were incubated with streptavidin beads (Sigma) for 90 min at 4°C. Following three washes, the samples were boiled with 2× sample buffer at 95°C for 10 min, resolved by SDS-PAGE, and immunoblotted. Antibodies used were as follows: GluA1 and GluK2 (Millipore), glyceraldehyde 3-phosphate dehydrogenase (GAPDH) and EGFR (Abcam), and ADAR2 (Sigma). Western blots were imaged and quantified using LI-COR Biosciences Image Studio software. The surface levels of GluA1, GluK2, and EGFR were normalized to their respective total levels to determine surface expression. Treated samples were normalized to their control samples.

### RNA Extraction and RT-PCR

RNA samples were extracted from DIV 14 hippocampal neurons after the stated treatments using the RNeasy Mini RNA extraction kit (QIAGEN) following the manufacturer’s protocol. 1 μg of RNA was used per condition and reverse-transcribed to cDNA using the RevertAid First Strand cDNA Synthesis Kit following the manufacturer’s protocol (Thermo Scientific). The following primers (spanning the M2 region of GluK2) were used, giving a PCR product of 452 bp: GluK2 F, 5′-GGTATAACCCACACCCTTGCAACC-3′; GluK2 R, 5′-TGACTCCATTAAGAAAGCATAATCCGA-3′. To determine the level of GluK2 RNA editing, BbvI (New England Biolabs) digestion was used (Bernard et al., 1999). Digestion of the PCR product was performed at 37°C for 2 hr. All of the digested product was run on 4% agarose gel, and the ethidium bromide-stained bands were imaged using a UV transilluminator and quantified using NIH ImageJ. To determine the level of editing, the following formula was used: (intensity of 376 [edited] / intensity of [376 (edited) + 269 (unedited)]) × 100. The band at 76 bp was used to determine equal loading.

## Author Contributions

A.J.E. performed the imaging experiments. S.G. performed the scaling and RNA editing experiments. K.A.W. helped design and make the constructs and provided practical advice and training. D.J.S. initiated the use of RUSH with A.J.E. in non-neuronal cells, and J.M.H. managed the project. A.J.E. and J.M.H. wrote the first draft of the paper. All authors made intellectual contributions and participated in editing the paper.

## Supplemental Information

Document S1. Figures S1–S6

Movie S1. SBP-SEP-GluK2 RUSH TIRF Imaging of Surface Exocytosis in HeLa Cells Combined, Related to Figure S1B

Movie S2. SBP-EGFP-GluK2 RUSH Colocalization with Dendritic ERESs, Related to Figure S2A

Movie S3. SBP-EGFP-GluK2 RUSH Colocalization with Dendritic Golgi Outposts, Related to Figure S2B

Document S2. Article plus Supplemental Information
