# Supplementary material for: Assembly, Secretory Pathway Trafficking, and Surface Delivery of Kainate Receptors Is Regulated by Neuronal Activity
Source: Cell Rep. 2017 Jun 20;19(12):2613–26. doi: 10.1016/j.celrep.2017.06.001 (PMC5489663; doi:10.1016/j.celrep.2017.06.001)
Supplement: Document S1. Figures S1–S6 [file mmc1.pdf]

Cell Reports, Volume *19*

Supplemental Information

Assembly, Secretory Pathway Trafficking, and Surface Delivery of Kainate Receptors Is Regulated by Neuronal Activity

Ashley J. Evans, Sonam Gurung, Kevin A. Wilkinson, David J. Stephens, and Jeremy M. Henley

Supplemental Information

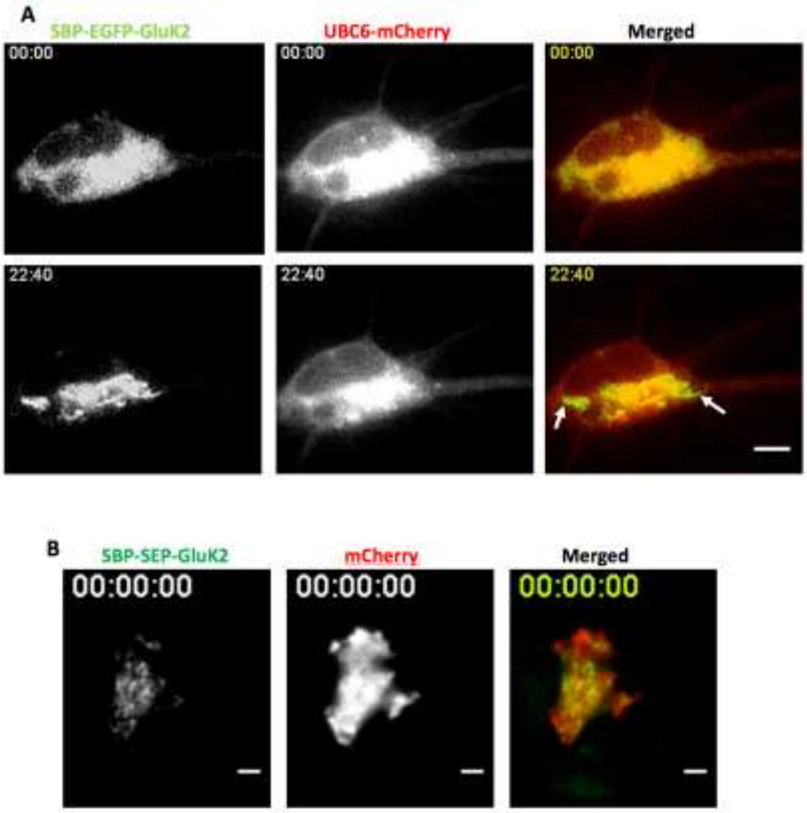

Figure S1. RUSH GluK2 receptor leaving the ER and exocytosing into the cell surface. Related to Figure 1. (see Supplemental Movie S1)

A) Primary hippocampal neurons were transfected with SBP-EGFP-GluK2 and an ER marker UBC6-mCherry (Wozniak et al., 2009). Frames were takien every 5 seconds after biotin addition. Still frames are shown demonstrating that before biotin there is colocalisation between SBP-EGFP-GluK2 and UBC6-mCherry and then a seperation after biotin addition (white arrows indicate seperation).

B) Movie from Figure 1D. HeLa cells were transfected for 20-24 hours with SBP-SEP-GluK2 and mCherry (to outline the cell). The bottom of the cell was found using TIRF imaging and biotin was added to release the 'hooked' receptor. Time is indicated in HR:MN:SC and split panels are shown for both SBP-SEP-GluK2, mCherry and a merged panel is also shown. Scale bars in all panels = 10µm.

Reference

Wozniak, M.J., Bola, B., Brownhill, K., Yang, Y.C., Levakova, V. & Allan, V.J. 2009. Role of kinesin-1 and cytoplasmic dynein in endoplasmic reticulum movement in VERO cells. *J Cell Sci,* 122**,** 1979-89.

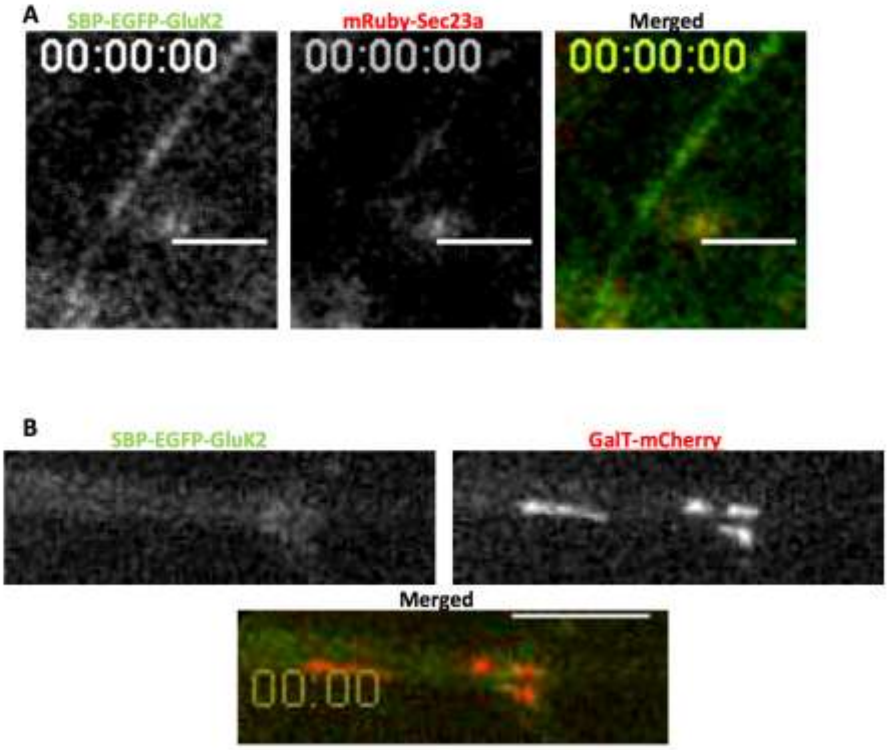

Figure S2: SBP-EGFP-GluK2 uses a local dendritic secretory pathway. Related to Figure 2.

A) Still image from the Kymograph shown in Figure 2C, please see Supplemental Movie S2. Primary hippocampal neurons were cotransfected for 20-24 hours with SBP-EGFP-GluK2 and mRuby-Sec23a to mark ER exit sites. Time is indicated in HR:MN:SC after the addition of biotin at the beginning of the movie.

B) Still image Kymograph shown in Figure 2G, please see Supplemental Movie S3. Primary hippocampal neurons were cotransfected for 20-24 hours with SBP-EGFP-GluK2 and GalT-mCherry to mark dendritc Golgi ouposts. Time is indicated MN:SC after the addition of biotin at the beginning of the movie.

Scale bars in all panels = 10µm.

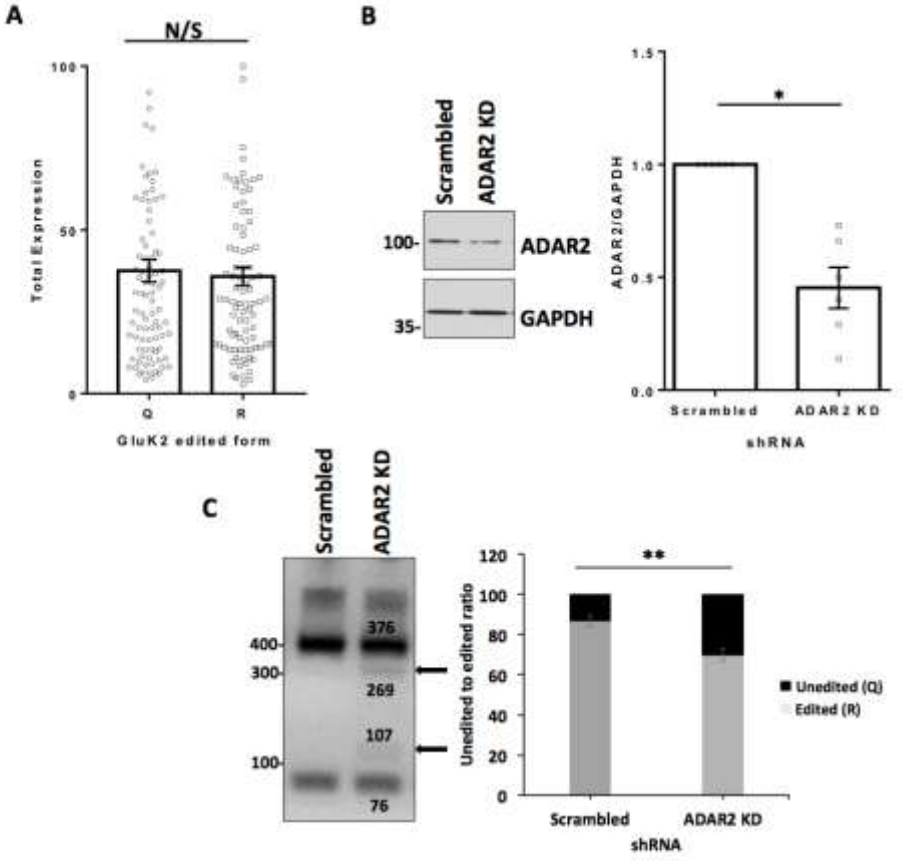

Figure S3: The GluK2 unedited (Q) and edited (R) are expressed at comparable levels and ADAR2 shRNA knocks down ADAR2 resulting in a decrease in GluK2 editing in primary neuronal culture. Related to Figure 3.

A) Total EGFP measurements (from Figure 3C, D) are compared from HeLa cells transfected for 20-24 hours with either SBP-EGFP-GluK2 (Q) or SBP-EGFP-GluK2 (R). This is to ensure there is no difference in the expression levels of the two different DNA constructs. 3 independent experiments and n=90. p>0.05, Welch's T Test.

B) Primary hippocampal neurons were infected with lentivirus expressing either scrambled or ADAR2 targeting shRNA, lysed and immunoblotted for ADAR2 and GAPDH. Graph shows quantification from 6 independent experiments. *=p<0.05, Wilcoxon matched-pairs signed rank test.

C) Primary hippocampal neurons were infected with lentivirus expressing either scrambled or ADAR2 targeting shRNA. Black arrows indicate unedited forms of GluK2. RT-PCR was performed on samples and digestion analysis of levels of unedited and edited GluK2 are displayed with quantification. **=p<0.01, Welch's T Test.

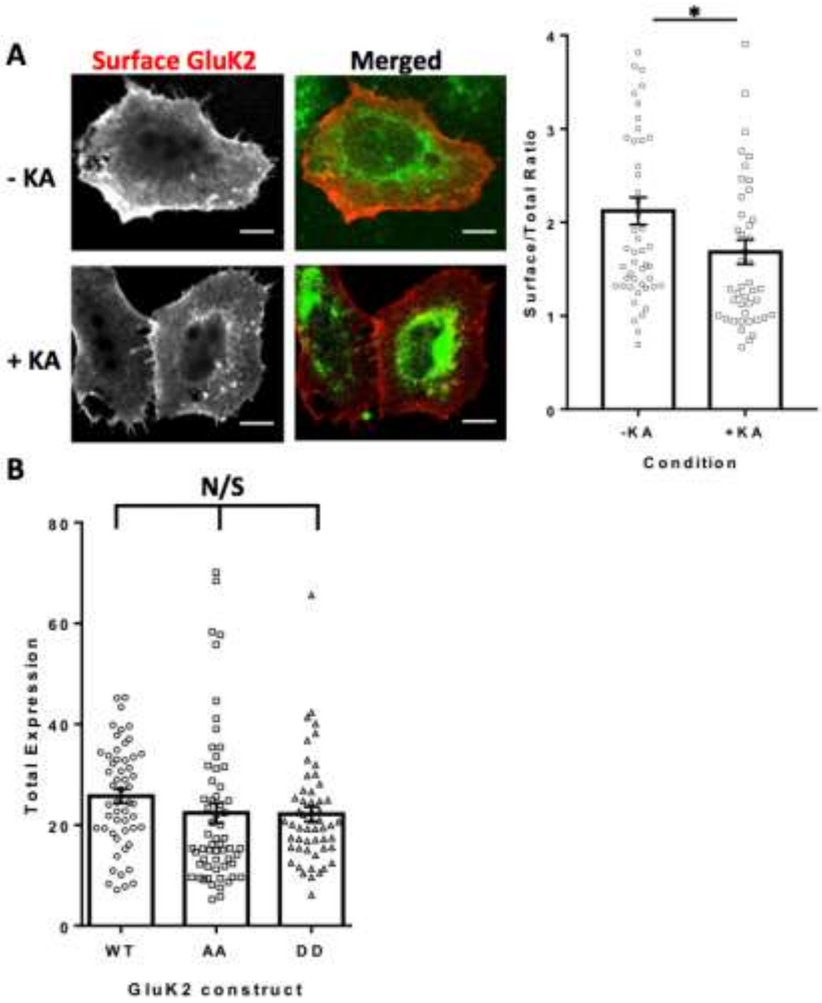

Figure S4: Kainate causes kainate receptor internalisation in HeLa cells and GluK2, GluK2 AA and GluK2 DD are all expressed at comparable levels. Related to Figure 4.

A) HeLa cells were transfected with SBP-mCherry-GluK2 (no hook, non RUSH variant) for 20-24 hours. Cells were untreated or exposed to kainate (100µM) and surface GluK2 labelled with Anti-SBP for 5 minutes. Cells were fixed in 4% PFA and not permeablised allowing labelling of just the receptors that remain at the cell surface and not the internilised population. Scale bars = 10µm. n=44-50, 2 independent experiments. *=p<0.05, Welch's T Test.

B) Total EGFP measurements (from Figure 4D, C) are compared from neurons transfected for 20-24 hours with SBP-EGFP-GluK2, SBP-EGFP-GluK2-AA or SBP-EGFP-GluK2-DD. This is to ensure there is no difference in the expression levels of the three different DNA constructs. 3 independent experiments, n=53-60. p>0.05, Welch's T Test.

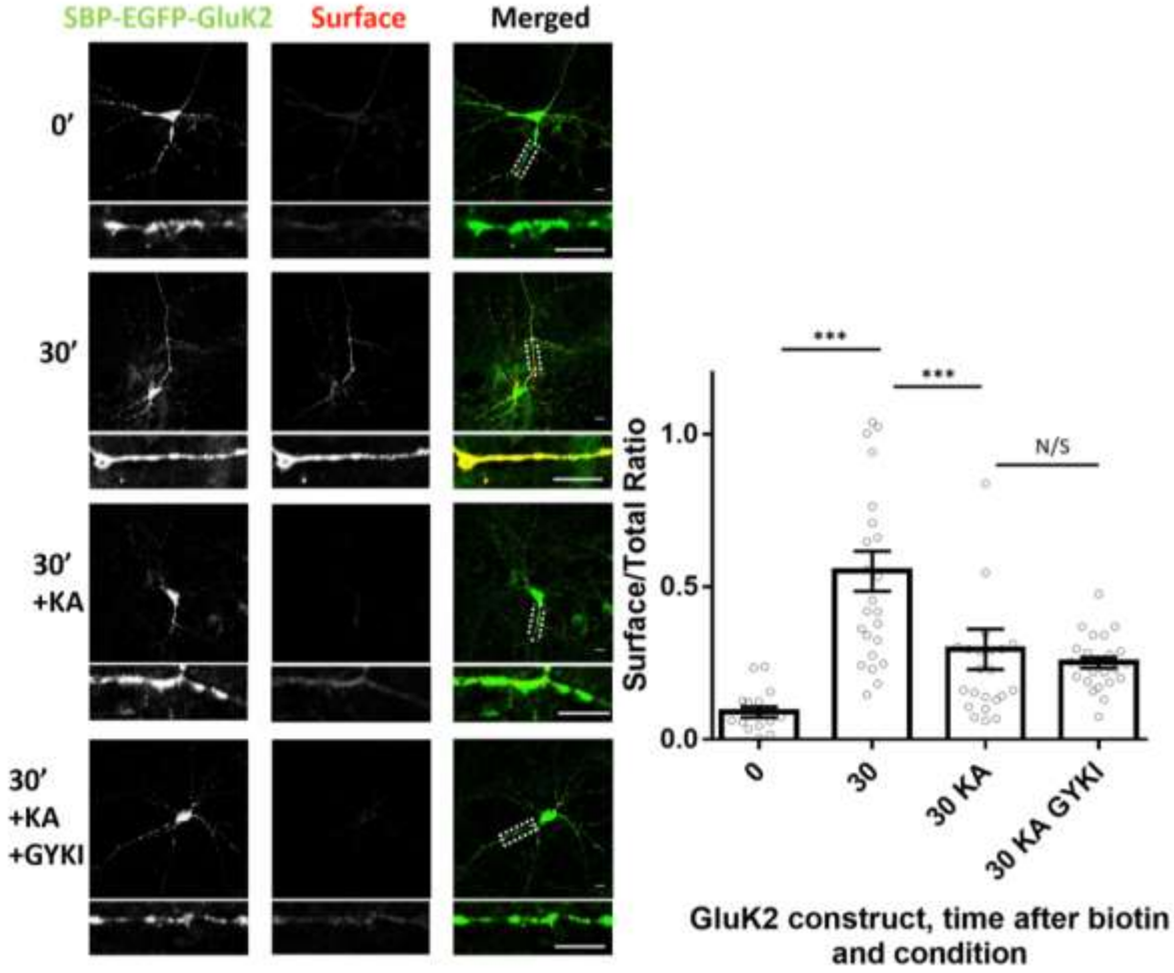

Figure S5: The transient kainate-mediated decrease in secretory pathway trafficking of de novo KARs is not mediated by activation of AMPARs. Related to Figure 5.

Primary hippocampal neurons were transfected with SBP-EGFP-GluK2 and released with biotin with or without a pre-biotin stimulation with 10µM KA or 10µM KA with 40µM GYKI-52466. KA still causes a decrease in *de novo* receptor surface delivery of KARs even in the presence of GYKI which inhibits AMPARs. ***=$p<0.001$, Welch's T Test. Scale bars = 10µm.

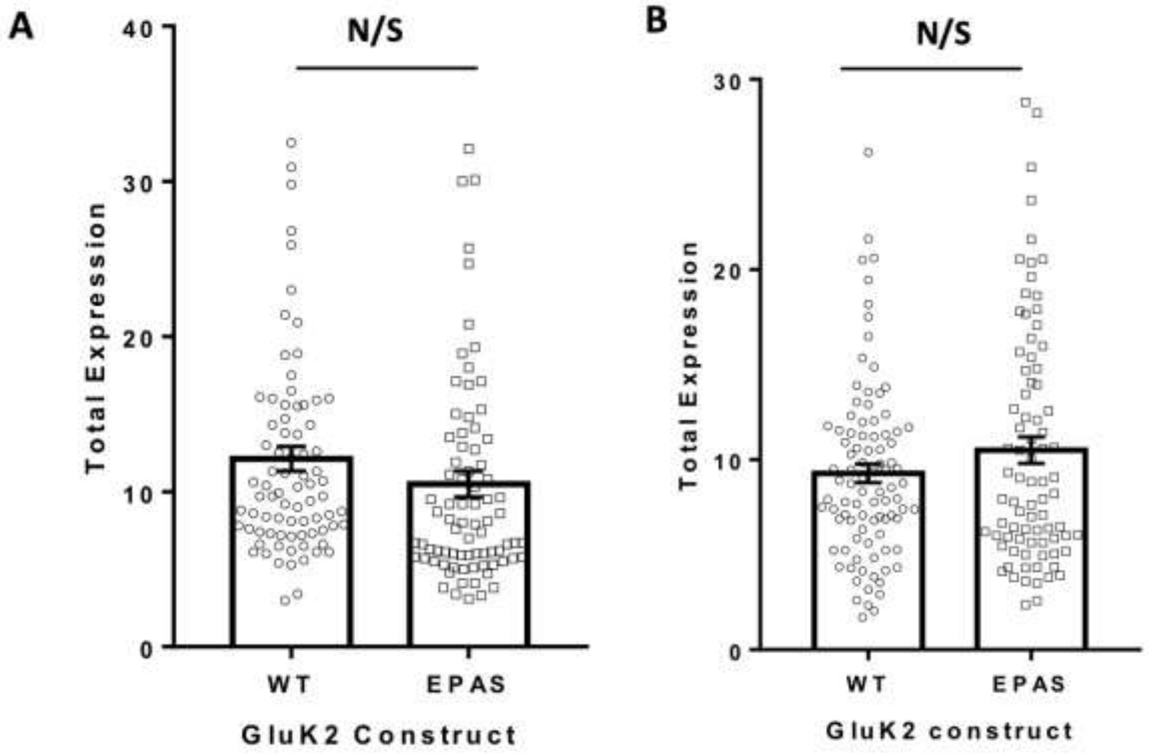

Figure S6: The GluK2 WT and EPAS mutant are expressed at comparable levels in both HeLa cells and neurons. Related to Figure 6.

A) Total EGFP measurements (from Figure 6A, B) are compared from HeLa cells transfected for 20-24 hours with either SBP-EGFP-GluK2 or SBP-EGFP-GluK2 EPAS. This is to ensure there is no difference in the expression levels of the two different DNA constructs. 3 independent experiments, n=80. $p>0.05$, Welch's T Test.

B) Total EGFP measurements (from Figure 6E, F) are compared from neurons transfected for 20-24 hours with either SBP-EGFP-GluK2 or SBP-EGFP-GluK2 EPAS. This is to ensure there is no difference in the expression levels of the two different DNA constructs. 4 independent experiments and n=82-92. $p>0.05$, Welch's T Test.
