# Supplementary figures and images for: Assembly, Secretory Pathway Trafficking, and Surface Delivery of Kainate Receptors Is Regulated by Neuronal Activity
Source: Cell Rep. 2017 Jun 20;19(12):2613–26. doi: 10.1016/j.celrep.2017.06.001 (PMC5489663; doi:10.1016/j.celrep.2017.06.001)

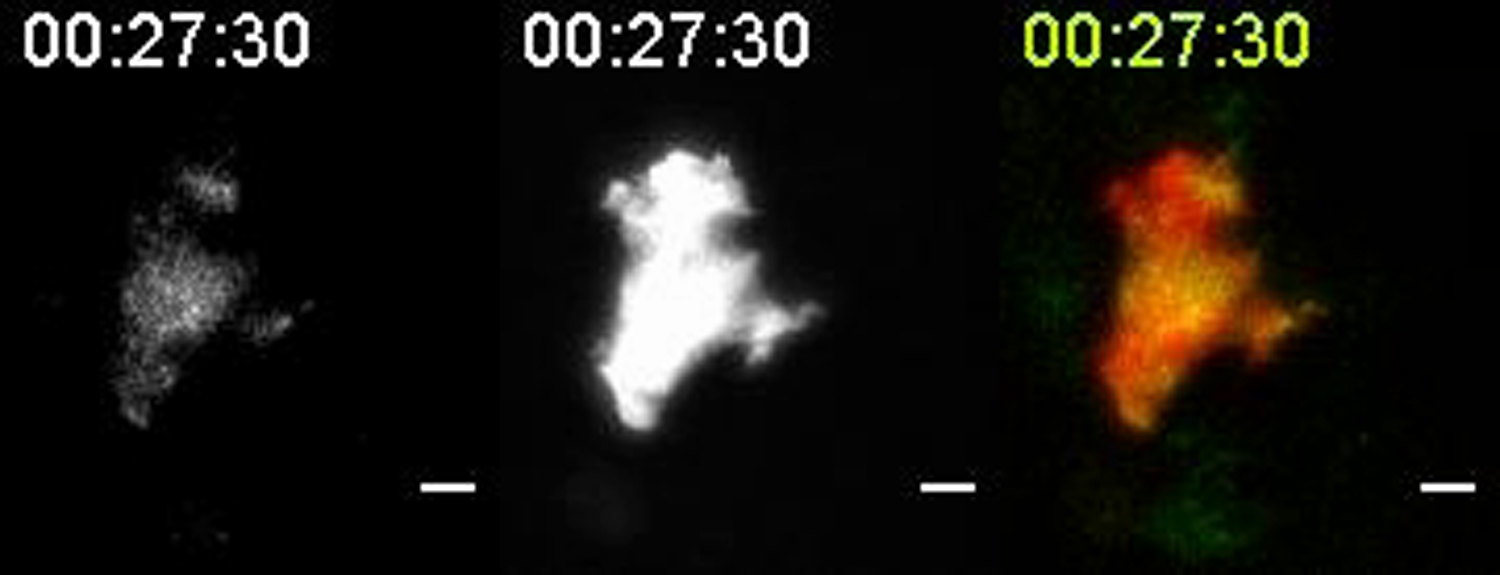

Supplement: Movie S1. SBP-SEP-GluK2 RUSH TIRF Imaging of Surface Exocytosis in HeLa Cells Combined, Related to Figure S1B [file mmc2.jpg]

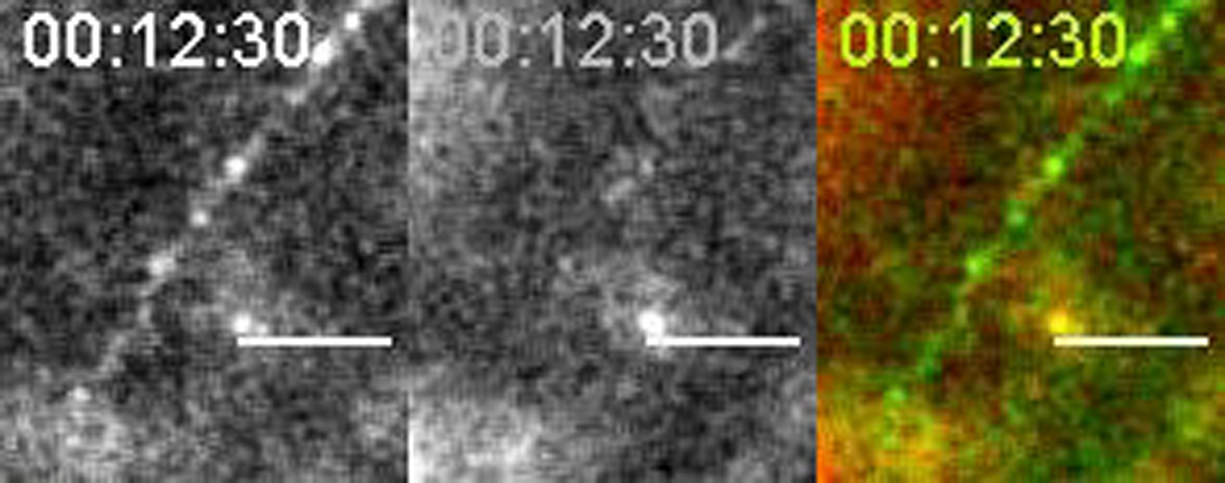

Supplement: Movie S2. SBP-EGFP-GluK2 RUSH Colocalization with Dendritic ERESs, Related to Figure S2A [file mmc3.jpg]

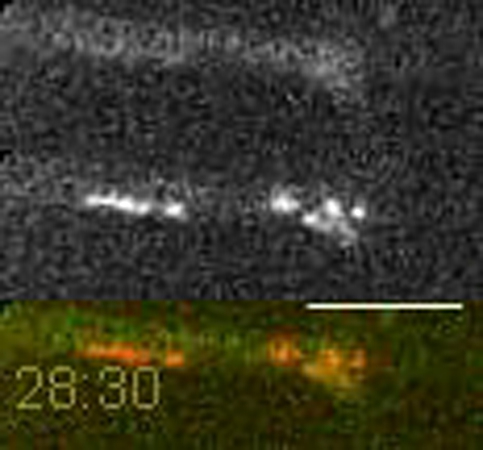

Supplement: Movie S3. SBP-EGFP-GluK2 RUSH Colocalization with Dendritic Golgi Outposts, Related to Figure S2B [file mmc4.jpg]
